# False Memories and Free Speech: Is Scientific Debate Being Suppressed?

**DOI:** 10.1002/acp.3285

**Published:** 2016-10-14

**Authors:** Bernice Andrews, Chris R. Brewin

**Affiliations:** ^1^ Royal Holloway University of LondonLondonUK; ^2^ University College LondonLondonUK

## Abstract

Commentators have raised important points, including the relative contribution of false beliefs versus false memories and the issue of how findings in the laboratory can be generalized to the real world, which we have addressed here. However, some of the commentaries misrepresent what we said, make criticisms that are unfounded, or imply that our article should not have been published in Applied Cognitive Psychology. We relate these responses to a more general literature on the suppression of unwanted scientific findings and suggest that the study of false memory would be better served by more openness to alternative perspectives. Copyright © 2016 The Authors Applied Cognitive Psychology Published by John Wiley & Sons Ltd

As experts who act both for the prosecution and defence in cases where adults' memories of childhood are at issue, we had been struck by the imprecise and sometimes misleading ways in which the literature on suggesting false childhood memories in the laboratory was often presented, both in the scientific literature and in court. It had been 21 years since the first experimental studies were published, yet no one in the field had produced a systematic review of this large and important body of research. We were surprised to find, however, when we previously submitted an almost identical version of our review to other scientific journals, that some of the referees' reports were exceptionally hostile. One of these referees, who has contributed to a commentary in the current issue, wrote, ‘So, more than 40 hours and eleven pages later, how should I sum up my review? This manuscript is basically a biased rant dressed up in pseudoscientific clothes… Their most crucial claims…are spectacularly inaccurate…this manuscript is not, and probably could not ever be, worthy of publication in a peer‐reviewed journal’. Another referee recommended rejection, commenting, ‘The authors appear to have some sort of political or social agenda that may be contributing to their dismissal or minimizing of the false memory findings’.

Readers can now judge for themselves how accurately these comments describe our paper. The current set of commentaries include many thoughtful and constructive responses, and we address some of the most important of these first. We believe that it is necessary to raise the issue of the suppression of scientific debate, however, because other commentaries, though they largely avoid the ad hominem attacks we received earlier, demonstrate other familiar rhetorical devices that are frequently employed to try to discredit scientific findings. We identify these in the second half of this commentary.

## Issues for the Field

Commentators have paid a huge amount of attention to our summary figure of 15%, representing the average number of participants who appeared to develop full false memories in implantation experiments. There are many potential ways to summarize the data, and we believe that it is healthy to debate this. In our paper, we attempted to show how investigators have tried to rate the complex subjective judgements by which participants decide if their mental experiences are more like beliefs or memories and whether apparent recollections are likely to correspond to a real event. The distinctions we used appear very similar to those articulated by Scoboria and Mazzoni (2016); although, as they note, because studies have not adopted a common framework, the links between concepts and measures used are sometimes approximate. Several commentaries noted an as yet unpublished ‘mega‐analysis’, based on eight memory implantation studies. Three of these studies appeared in the column corresponding to ‘full false memory’ and five in the column corresponding to ‘false memories partially meeting full criteria’ in table 4 of our review. Based on re‐rating the original memory transcripts, Scoboria et al. (2016) concluded that 11% of the false memories were ‘substantial’ (their most conservative category) and a further 9% were ‘complete’ (their next most conservative category). It is striking that despite the methodologies being entirely different, the prevalence of 15% we estimated based on our most conservative category falls midway between estimates based on Scoboria et al.'s two most conservative categories.

This convergence greatly strengthens confidence in the conclusions of ourselves and Scoboria et al. (2016). The way both sets of authors operationalized their concepts has the merit of being explicit, so that the effect of different assumptions can be tested. The important point, it seems to us, is that in a therapeutic or courtroom context, it is of the utmost importance to be aware of the range of possible subjective states, rather than using a generic label of ‘false memories’ to cover widely differing experiences. The data show clearly that more stringent definitions of what constitutes a false memory lead to lower rates.

Some commentators (Lindsay & Hyman, 2016) have objected to our reference at one point to 15% as an ‘upper bound’, pointing out that rates will fluctuate under different conditions. It is true that investigator‐determined rates of what we have defined as full false memories were as high as 25–26% in a couple of studies. Our estimate factors in concerns about the file drawer problem (Becker‐Blease & Freyd, 2016; Pezdek & Blandon‐Gitlin, 2016), evidence we identified that investigator ratings probably overestimate false memory rates relative to participant self‐ratings, and the likelihood that some events assumed false by investigators could in fact be true. Ultimately, this is a matter of judgement. However, we cannot agree that ‘depending on the criteria one uses, full false memory creation can range from 15% up to 46%’ (Otgaar, Merckelbach, Jelicic, & Smeets, 2016). Given that the higher figures they mention are based on some experiences that participants do not believe correspond to an actual memory, we struggle to understand how this range can possibly provide ‘a more reliable estimate of the potential to implant full false memories for entire events than the lower bound percentage’.

A point made by Scoboria and Mazzoni (2016) is that in our focus on full false memories, we have underestimated the significance of false beliefs. As noted by Pezdek and Blandon‐Gitlin (2016), we agree that we have largely followed the field in concentrating on the implantation of full false memories as this has the greatest relevance for situations where therapists may create memories of childhood trauma. A few of the false feedback studies we review do show some behavioural effects of inducing false beliefs, although these are largely confined to small increases or decreases in the selection or consumption of certain foods. Nevertheless, we agree with Scoboria and Mazzoni that increasing a false belief of having been abused in childhood is equally reprehensible and could have damaging consequences to the individual and others. Smeets, Merckelbach, Jelicic, and Otgaar (2016) further suggest that a false belief might be sufficient to start a legal proceeding. It would be valuable to be able to go beyond the largely anecdotal evidence available at this point. However, we agree that on occasion, it might certainly be enough to start an investigation and could have very negative consequences, even though its chances of going to court are most likely very slim.

The issue raised by most commentators concerns how to generalize the results of these experimental studies to the real world. As McNally (2016) correctly notes, this is not at all straightforward. He discusses conditions likely to increase false memories occurring among patients in therapy, including a belief in the existence of repressed memories, a belief that they can cause symptoms, and a belief in the power of techniques suggested to retrieve them, as well as certain cognitive styles. In our article, we discussed which aspects of therapy might be comparable to processes examined in the experimental studies and might have damaging consequences, as well as ways in which therapy differs from these studies. It is notable, however, that several of the commentators assume that the experimental techniques are weak versions of what may happen in the real world (Lindsay & Hyman, 2016; Nash, Wade, Garry, Loftus, & Ost, 2016; Smeets et al., 2016), involve ‘minor and rapid manipulations’ (Scoboria & Mazzoni, 2016), or express astonishment that they result in any false memories at all (Nash et al., 2016).

This opinion that the memory implantation studies involve weak manipulations and must therefore underestimate real‐world dangers is worth considering in more detail. Other false memory experts (Hyman & Pentland, 1996), in contrast, have thought that the studies involved very high levels of demand on college student participants. One factor that has not been brought out clearly is that the experimental studies involve deliberate deception from trusted and authoritative family members *often accompanied by other specific and seemingly incontrovertible corroborative evidence*. In our view, deception involving family witnesses and doctored photos for unremembered events is not a trivial or mild intervention. It is an integral part of all implantation studies and goes beyond the therapeutic analogues of strong suggestion and pressure to remember apparent in all false memory paradigms. It is unclear to us why it should ever have been considered as an analogue of what goes on in the therapist's office.

Given the weak effects of suggestion obtained in the other paradigms where strong deception is not used, we suspect that it is the powerful combination of deception and pressured suggestion that is the main driver of false memories in the laboratory. Moreover, deception is likely to be related to false beliefs and acceptance that are often included in investigator‐based assessments. Although therapists may repeatedly suggest that events such as childhood abuse have happened, they are never in this position of being able to confirm events *because they themselves were there*. As a result, participants in the experimental studies may be *more* inclined to imagine the suggested events, and in some cases, to develop full false memories.

The same ambiguity surrounds anecdotal claims about what happens in therapy and the assumption that memories are recovered because of inappropriate therapeutic practice (Nash et al., 2016; Smeets et al., 2016). In many cases, it appears that memories of abuse are recovered prior to therapy or in the absence of suggestive techniques (Andrews et al., 1995; Andrews et al., 1999). But among therapists, knowledge of how memory works is far from perfect, particularly for those who are not qualified as psychologists (Brewin & Andrews, 2014), and this lack of awareness can have very damaging consequences. In cases we have reviewed, we have seen examples both of inappropriate practice leading to highly dubious patterns of recall and to a much more spontaneous and limited type of memory recovery. We do not believe that at this point, it is reasonable to make any claims about whether the rate of false memories is likely to be higher in the laboratory than the clinic or vice versa. What we would like to see in the future is a more nuanced appreciation of these issues and for experts to desist from making misleading claims about the relevance of the laboratory to the real world.

## Suppression of Alternative Perspectives

Here, we identify some examples of rhetorical or misleading attempts to discredit our findings and conclusions, similar to those we have encountered during the review process.

### Pejorative language

Our message (that experts should be wary when informing the courts about this literature) was described by Smeets et al. (2016) as ‘naïve’. Nash et al. (2016) refer to our paper as ‘a flawed opinion piece masquerading as a peer reviewed article’ and suggest that it ‘ignores the facts (about memory fallibility) so blithely that it seems more suited to being called an op‐ed piece than a peer‐reviewed “research article”’. This is intriguing, considering that it is being published alongside commentaries from many of the leading experts in the area. It is striking that none of these commentators appear to have discovered flaws in the comprehensiveness of our search strategies or in the extraction of and summaries of the data. A number of them appear to agree with our conclusions, and some even suggest that they have expressed similar opinions to ours in earlier publications (Lindsay & Hyman, 2016).

### Misrepresentation

A commonly used tactic in trying to suppress alternative opinions is to criticize the authors for writing something that is clearly fallacious or ill‐considered even though they did not in fact do this. Some of the most egregious examples of this tactic are described here. Lindsay and Hyman (2016) claimed repeatedly that we attempted to establish an upper limit on the probability that adults who were not abused as children can be led to believe that they were, which clearly could never be inferred from the experimental evidence. Our conclusions made no reference to this real‐world scenario: ‘*From our review of* (*memory implantation*) *studies*… *the upper bound would seem to be about 15% of participants*’. Nash et al. (2016) claimed that we drew conclusions about the fallibility of memory in general, not just about suggesting memory for childhood events, which enabled them to criticize us for not including other kinds of evidence. But our review is explicitly focussed on false childhood memories and at no point seeks to draw wider conclusions; for example, we state that ‘*it cannot be concluded that false memories of childhood events possessing these characteristics are common*, *that they are easy to suggest or implant or that the majority of individuals are susceptible to them*’. Another misrepresentation was that we downplayed the potential influence of therapists, suggested by Lindsay and Hyman (2016), Smeets et al. (2016), Scoboria and Mazzoni (2016), and Nash et al. (2016). On this issue, we invite readers to look at our conclusions and reassure themselves that we have considered the role of therapists in a detailed and balanced way. In addition, we were accused of making out that the importance of false memories had been exaggerated. For example, Nash et al. claimed that we imply that ‘scientists are making a mountain out of a molehill’. Our actual words expressed the very opposite opinion: ‘*The fact that susceptibility to false memories appears to be lower than has often previously been suggested does not diminish in any way the significant implications* (*of this literature*) *for the courtroom and the need to consider the extremely damaging consequences that might ensue*’. Finally, Scoboria and Mazzoni (2016) complained that we imply that people are not influenced by imagination inflation procedures, when we clearly stated that ‘*the data indicate fairly conclusively that on average the imagination paradigm increases participants' beliefs that events*, *originally perceived as unlikely to have happened*, *are more likely to have occurred than they first estimated*’.

### The ‘straw man’ argument

This is the criticism that we identified mistaken views about the literature that nobody in fact holds. Lindsay and Hyman (2016) point out that some memory researchers have previously articulated the arguments that we ‘appear to be offering as novelties’. We agree, but for us to be setting up a ‘straw man’ requires that these insights have become generally accepted and part of the way researchers discuss the results in this field. If this was the case, then it is not clear why our article attracted such hostile responses. Although some of the commentaries do broadly accept our conclusions, others—particularly those by Nash et al. (2016), Otgaar et al. (2016), and Smeets et al. (2016)—do not appear to accept any of them as valid. Lindsay and Hyman ask, ‘Who could dispute that use of the term “false memory” without qualification or explanation has the potential to be misleading?’ As we note below, however, the commentary by Otgaar et al. (2016) provides a classic example of the dangers of using the term ‘false memory’ to include numerous quite different phenomena.

### Flaws in our methods

The main criticism, made in only one of the commentaries, is that our choice of literature was selective and that we omitted important material Otgaar et al. (2016). But, as these authors themselves admit, none of the studies they mention actually involved memory for childhood events, which was the subject of our review. If the charge is that we omitted irrelevant material, then we will have to plead guilty. Citing misinformation studies for nonautobiographical events, Otgaar et al. (2016) argue that the high confidence found in these ‘implanted false memories’ contradicts our conclusions, thus illustrating how use of the term ‘false memory’ can be narrowed or broadened to suit a particular rhetorical purpose (Becker‐Blease & Freyd, 2016). Of course, as Lindsay and Hyman (2016) rightly note in their commentary, ‘It is easier to engender false reports of trivial details in an inconsequential event than to create false reports of significant life experiences’. Finally, Otgaar et al. also criticize us for including literature on their crashing memory paradigm which does not involve childhood events. Readers will note that we cited this study in an introductory section and that it did not figure in our data tables.

### Flaws in our description of study results

Lindsay and Hyman (2016) argue that we overstate our case by saying, ‘even when clear memories were identified by the investigators, participants' confidence in them was below the scale midpoint’. They say that this is true in a number of studies but not all, citing Lindsay et al (2004) as showing that ‘self‐ratings of confidence, reliving and remembering for the suggested event…were equivalent to and in some cases directionally greater than their ratings for true events…’. This is misleading on two counts. First, in Lindsay et al. (2004), the only rating of a suggested event that was higher than the midpoint was a measure of autobiographical belief (belief the event happened), not the confidence in memory to which we referred. Second, what they are comparing here are ratings for the subset of participants who had memories of the suggested event versus equivalent ratings for true events in the entire sample, whether they could remember the events or not. Like‐for‐like comparisons show a clear advantage in favour of true memories. Lindsay and Hyman also cite Hyman and Pentland (1996) as showing that self‐report ratings of false memory image clarity and confidence were equivalent to ratings of *recovered* true memories (our italics). Readers should note that over 75% of true memories were always remembered, and study ratings of confidence in memory and imagery clarity for these, averaged across conditions, were consistently higher than for false events.

Scoboria and Mazzoni (2016) accept that some memorial reports judged to be recollections by observers may not be based on true recollections but assert that what we do not consider is that some events that are subjectively experienced as recollected may not be perceived by observers as such. Citing Otgaar and colleagues (Otgaar, Scoboria, & Smeets, 2013), they state, ‘the issue of miscommunication about the subjective basis of memory reports runs in both directions’, and therefore our observation ‘cannot solely be used to reduce estimates of memory formation rates’. However, an analysis of the figures in table 1 of Otgaar et al. (2013) shows that in 41% of instances where observers rated a memory for the suggested event, the participants themselves did not report such a memory. When observers rated no memory for the event, only 9% of participants disagreed, a large difference significant by McNemar's test (*p* < .01). This clearly suggests that rating discrepancies between researchers and participants are a reason to reduce estimates of memory formation.

Scoboria and Mazzoni (2016) also suggest that we could have made more of the influence of moderator variables and included estimates of the size of effects for factors that are associated with higher likelihoods of false belief and false recollection. The only one they suggest is event plausibility, which is already well‐established (Pezdek & Blandon‐Gitlin, 2016). We have discussed in the paper why we did not think that the data lent themselves to meta‐analysis; the small number of relevant studies poses additional problems for moderator analyses, which are not recommended in the absence of sufficient statistical power (Hedges & Pigott, 2004).

## Conclusions

Why do we not content ourselves with pointing out the errors and misrepresentations in the commentaries but additionally suggest that they amount to an attempt to suppress scientific debate? Partly, this is because of the discrepancy between the extreme hostility of some of the original reviews and the questioning of our motives, coupled with the absence of any coherent scientific critique. It is notable that the objections appear to be more about the implications of the data than the data themselves. Whereas we have attempted to give a balanced perspective on how the data may be relevant to therapists' activities, our critics appear only to be concerned with the consequence that therapist‐induced false memories might be perceived as less common or less important than what was previously thought. In reality, there are other significant dangers; for example, that exaggerating the role of false memories might lead to the accounts of genuine abuse survivors being wrongly dismissed. It is not ‘naïve’ (Smeets et al., 2016) to be able to consider multiple perspectives. One consequence of the narrow focus to date is that alternative scenarios such as perpetrator‐induced alterations in recall of childhood experiences have been almost entirely neglected (Becker‐Blease & Freyd, 2016).

The clearest indication that there has been an attempt to suppress our data is contained in the final section of the commentary by Nash et al. (2016), in which they all but state that our article should not have been published because it did not go through a conventional peer review process with Applied Cognitive Psychology. We believe that this and the other hostile responses we have received are part of a more general pattern that can be discerned across a number of controversial areas of science. It is recognized that theoretical rigidity and lack of open dialogue are a particular problem for theories that are rooted in public policy or social morality advocacy goals. As Ferguson (2015) notes (p. 532), ‘This occurs when scholars become emotionally invested in a theoretical perspective. Inconclusive data may be interpreted as supportive of the theory and nonsupportive data ignored, criticized, or suppressed’. Likewise, partisans assume that their own views of the world are objective and infer that subjectivity (e.g. due to personal ideology) is the most likely explanation for their opponents' conflicting perceptions (Keltner & Robinson, 1996). Empirical evidence supports the fact that reviewers' evaluations are biased by whether the study's results favour their own theoretical position (Mahoney, 1977).

In order to avoid the possibility that data which contradict reviewers' assumptions are suppressed, it has been recommended that all articles and reviews be published, separating the review process from the publication decision. Our experience suggests that in some controversial areas, this approach is necessary and that journal editors often fail to challenge or correct a flawed review process. We therefore applaud the editors of Applied Cognitive Psychology for making our data and arguments available and encouraging a wider debate. The views of Nosek and Bar‐Anan (2012) appear to be particularly relevant to the study of false memories: ‘Truth emerges as a consequence of public scrutiny—some ideas survive, others die. Thus, science makes progress through the open, free exchange of ideas and evidence’ (p. 217).
